# Relationship between static and dynamic balance in 4-to-5-year-old preschoolers: a cross-sectional study

**DOI:** 10.1186/s12887-024-04747-6

**Published:** 2024-05-09

**Authors:** Ruqiang Liu, Juan Yang, Feifei Xi, Zichun Xu

**Affiliations:** 1 Suzhou Early Childhood Education College, Suzhou, Jiangsu Province China; 2 School of Physical Education, Shanghai University of Sport, Shanghai, China; 3 Suzhou Institute of Trade and Commerce, Suzhou, Jiangsu Province China

**Keywords:** Preschool children, Static balance, Dynamic balance, Angular velocity modulus

## Abstract

**Background:**

Balance is crucial for physical development in preschool children. Exploring the relationship between different types of balance can help understand early physical development in children. Currently, research is mostly focused on the relationship between different types of balance in the adult population and lacks exploration of the preschool population. The aim of this study explored the relationship between static and dynamic balance in preschool children aged 4 to 5 years.

**Methods:**

A total of 128 preschool children between the ages of 4 to 5 years were selected. The following tests were conducted as they wore inertial sensors detecting their centers of mass (COM): T1, standing with eyes open; T2, standing with eyes closed; T3, standing with eyes open on foam; T4, standing with eyes closed on foam; and T5, walking on the balance beam. Static balance was measured by the angular velocity modulus (*ω*_−T1_–*ω*_−T4_) of the shaking COM, as well as the pitch angle (*θ*_−T1_–*θ*_−T4_) and roll angle (*φ*_−T1_–*φ*_−T4_) indicators in T1–T4 testing. Dynamic balance was measured by the time (*t*) and angular velocity modulus (*ω*_−T5_), as well as the pitch angle (*θ*_−T5_) and roll angle (*φ*_−T5_) indicators in the T5 test. The Pearson product-moment correlation coefficient was used to test the correlation between static and dynamic balance indicators.

**Results:**

There is no correlation between *ω*_−T1_–*ω*_−T4_ and *t* (*P* > 0.05), while *ω*_−T1_–*ω*_−T4_ and *ω*_−T5_ (*r* = 0.19–0.27, *P* < 0.05) and *ω*_−T1_–*ω*_−T4_ and *θ*_−T5_, *φ*_−T5_ (*r* = 0.18–0.33, *P* < 0.05) were weakly correlated. There is no correlation between *θ*_−T1_–*θ*_−T4_, *φ*_−T1_–*φ*_−T4_ and *t* (*P* > 0.05), while *θ*_−T1_–*θ*_−T4_, *φ*_−T1_–*φ*_−T4_, and *θ*_−T5_, *φ*_−T5_ were weakly correlated (*r* = 0.01–0.28, *P* < 0.05).

**Conclusions:**

The relationship between static and dynamic balance in preschool children aged 4–5 years is weak. Static and dynamic balance in children needs to be intervened separately for the development of children.

## Background

Balance is an important aspect of the health of preschool children and a fundamental condition in their motor development [1]. Based on different standards, balance capacity can be divided into two (static and dynamic balance) [2], three (static, dynamic, and symmetric balance) [3], or four types (static, dynamic, active, and reactive balance) [4]. In recent years, the relationships between different types of balance have garnered attention. Researchers have explored the relationship between static and dynamic balance among people of different ages, such as children over 7 years old, teenagers, middle-aged, and older adults [5], and of different occupational groups, such as professional sports, dancers, and other professionals [6, 7]. At the same time, the correlation between the four types of balance in people over six years old has also been explored: static, dynamic, reaction, and active balance [8]. The results obtained were similar; that is, there is little to no correlation between the different types of balance abilities. However, there is still a lack of correlation research on the different types of balance in children under six years of age. Before the age of 6, balance develops rapidly, especially at 4–5 years of age when balance control shifts from visual dominance to the same proprioceptive dominance mode as in adults. At this time, balance has notable characteristics, the fastest development speed, and greatest variability of all the stages in life [9]. Therefore, it is difficult to generalize the research results of people over 6 years old to the preschool children group. Given that the targeted samples of previous studies were mostly children over 7 years, adults, and older adults, it is important to investigate the relationship between static balance and dynamic balance in preschool children under 6 years old. A better understanding of their association helps to give an insight into the development mechanisms of various balance abilities in the early years. Meanwhile, such an understanding might also assist physical activity and health professionals, teachers, and coaches in implementing precise balance intervention programs for preschool children.

In recent years, with the development of Micro-Electro-Mechanical Systems (MEMS), wearable sensors have gradually been applied for the detection of human balance [10]. Wearable sensors can accurately measure the static and dynamic balance of preschool children under different sensory conditions or sports states and can make up for the many defects in the “gold standard” plantar pressure test of balance [11]. The plantar pressure test evaluates human posture by collecting center of pressure (COP) data. COP is different from center of mass (COM), and COM is more suitable as an indicator of changes in body posture [12]. This study used wearable sensors in combination with a modified clinical test of sensor interaction and balance (mCTSIB), and a balance beam walking test was employed to explore the correlation between static and dynamic balance in preschool children aged 4–5 years.

The wearable sensor combined with the mCTSIB can measure the shaking speed and amplitude of the COM under normal conditions, as well as shielding vision, interfering with proprioception, and sensor conflict. Combined with the balance beam test, it can measure the speed of walking on the balance beam, and the shaking speed and amplitude of the COM when walking on the balance beam. This study will, therefore, show the correlation between static and dynamic balance in preschool children from multiple perspectives through indicators such as shaking speed and amplitude of the COM under various sensory conditions and different movement states. This research aimed to further understand the early physical development of children and provide a reference for promoting balance and motor development in children.

## Methods

### Sample

A total of 216 children from all six middle classes of a public kindergarten in Suzhou, China, as the sampling frame. The participants were stratified according to sex, and a random number generator [13] was used to select 64 males and 64 females, leaving a total of 128 participants. Cohen’s DI was 0.8, the effect size was moderate (0.30), *α* was 0.05, and the required sample size for the product distance correlation test was 85 [14]. The sample size in this study met the research requirements, and basic information about the sample is shown in Table 1. The inclusion criteria were physical integrity, absence of mental illness, normal vision, and guardian consent to participate in the study. The exclusion criteria were cognitive dysfunction, visual impairment, autism, and medical history within one week.

**Table 1:** Basic information about the participants

Group	Number	Age (month)	Height (cm)	Weight (kg)	BMI (kg/m^2^)
Male	64	59.89 ± 3.45	112.89 ± 4.32	20.41 ± 2.72	15.97 ± 1.38
Female	64	59.81 ± 3.86	112.24 ± 5.18	19.95 ± 3.12	15.78 ± 1.66
Total	128	59.85 ± 3.65	112.56 ± 4.76	20.18 ± 2.92	15.87 ± 1.53

### Instruments

1. Wearable sensor: This study used an MPU-9250 wearable sensor (Motion TrackingTM series product, TDK InvenSense, Sunnyvale, California, USA) for the measurements. It can measure the angular velocity and acceleration of object motion in real-time and has been widely used in fields, such as attitude detection and health-intelligent equipment [15]. During the testing process, the sensor transmitted real-time data to the upper computer using Bluetooth, and the upper computer quickly solved the real-time motion posture of the sensor using calibration algorithms. During the test, the parameters were set as follows: gyroscope range of 2000 dps, acceleration sensor range of 16 *g*, magnetic range of 8 Gauss, and data transmission rate of 200 Hz.
2. Foam pad: This study used the pad (Xiyuan Co., Ltd., Zhengzhou, China) was of the specifications 50 cm × 50 cm × 8 cm, with a density of 40 kg/m^3^. This foam pad was smooth and uniforma.
3. Balance beam: This study used the balance beam (Mofang Co., Ltd., Qingdao, China) recommended in the Handbook of Chinese National Physical Fitness Measurement Standards (Children’s Part). To eliminate the fear of the participants, the height of the balance beam was adjusted from 30 cm to 5 cm, leaving the final specifications of 300 cm × 10 cm × 5 cm, as per previous studies [16].

### Measurement method

**Fig. 1:**
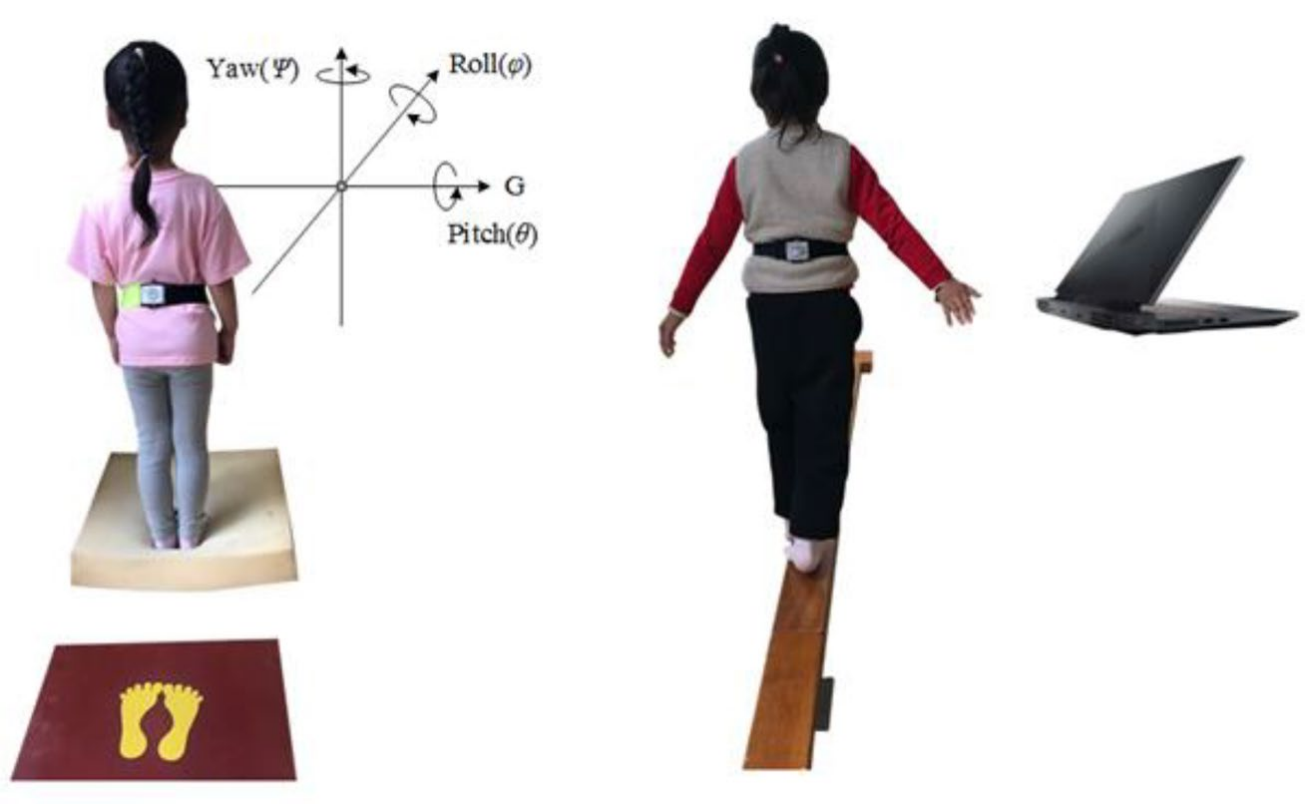
Balance test scenario diagram

The sensor was placed on the T12 position (Thoracic Vertebra 12) of the participant for testing as it can reflect the shaking of COM in the body of preschool children (Fig. 1) [17]. The static balance test project was the mCTSIB, which included (T1) standing with both feet open in place: standing with both feet close to the ground, with both eyes facing forward, trying to maintain body stability; (T2) standing with eyes closed and feet in place: both eyes closed based on the conditions of T1; (T3) standing with both feet open on the foam pad: standing with both feet close to the foam pad, with both eyes facing straight ahead, and striving to maintain body stability; and (T4) standing with eyes closed and feet on the foam pad: eyes were closed after following the conditions of T3. During the test, the participants were required to focus on a picture book placed in front of them (except when their eyes were closed), with their arms hanging down naturally on both sides of the body, their shoes off, and their front soles and heels placed together. If the participant speaks, coughs, turns their head, or scratches their head during the test, the item was retested. Each project was tested for 35 s, and, according to previous research recommendations [18], the first 5 s of data were excluded and data from the 5th to 35th s were included in the statistical analysis. The dynamic balance test (T5) involved walking on the balance beam; the participant first stood at one end of the balance beam, started after hearing the command, and stopped when any foot reached the end. It was necessary to remove shoes and walk alternately on the balance beam. During data extraction, data from the 2nd s to the end of walking on the balance beam were included in the statistical analysis. The participants completed the tests in the order of T1 to T5, with a break of approximately 30 s between each test item.

The testing dates were March 22–31, 2021, between 8 am and 11 am. During the testing period, the room temperature was 13–17 ℃, and the relative humidity was around 85%.

### Analysis indicators

1. Angular velocity: Angular velocity is a commonly used indicator for analyzing human posture [10] and reflects the speed of body shaking. This study extracts the angular velocity modulus (*ω*) of the COM, which reflects the rate of body shaking. *ω* is calculated using the angular velocity data of the three axes of the human body through Eq. (1) [19] which represents the overall shaking velocity of the human body in the three axes. When analyzing static balance, *ω*_−T1_, *ω*_−T2_, *ω*_−T3_, and *ω*_−T4_ were calculated as the analysis indicator by obtaining angular velocity data under different sensory conditions of T1–T4, as per existing research [11]. During the analysis of dynamic balance, the time taken to walk the balance beam was extracted, with ω as an analytical indicator. When extracting indicators, in order to avoid the impact of the speed of walking on the balance beam on the angular speed, as per previous studies [20], the *ω* value was divided by the speed of walking on the balance beam (*ω*_−T5_); this is taken as the index of dynamic balance analysis.

1 $$\omega =\sqrt{{\omega }_{x}^{ 2}+{\omega }_{y}^{ 2}+{\omega }_{z}^{ 2}}$$

Among these values, *ω* is the angular velocity modulus, while *ω*_*x*_, *ω*_*y*_, and *ω*_*z*_ represents the angular velocity data of the *x*-axis, *y*-axis, and *z*-axis, respectively.

(2) Attitude angle: The attitude angle displays the amplitude of body shaking. The commonly used for human posture angle is pitch (*θ*), roll (*φ*), and yaw (*ψ*). Among these, the change in *θ* represents the amplitude of the body’s longitudinal movement, the change in *φ* indicates the amplitude of the body’s transverse direction, and the change in *ψ* represents the extent to which the body rotates to the left and right. Since the *ψ* error is relatively large and difficult to calculate [21], this study only analyzes changes in *θ* and *φ*.
(3) Time taken to walk on the balance beam: As per the Handbook of Chinese National Physical Fitness Measurement Standards (children’s part), the time (*t*) of walking on the balance beam was selected as one of the indicators to investigate the dynamic balance of the participants. The shorter the time, the better the dynamic balance.

### Statistical analysis

SPSS software (version 25.0) was used for the statistical analysis. First, the Shapiro–Wilk test was performed on the data from each group. Lg10 conversion was performed when data did not conform to a normal distribution. The data was presented in the form of$\stackrel{-}{x}$±*s*. The Pearson product distance partial correlation coefficient was used to evaluate the correlation between static and dynamic balance after controlling for age. The correlation coefficient *r*<| 0.10 | indicates no correlation, | 0.10 | ≤ *r*<| 0.30 | indicates weak correlation, | 0.30 | ≤ *r*<| 0.50 | indicates moderate correlation, and *r* ≤ | 0.50 | indicates strong correlation [13]. The significance level was set at *α* = 0.05.

## Results

**Fig. 2:**
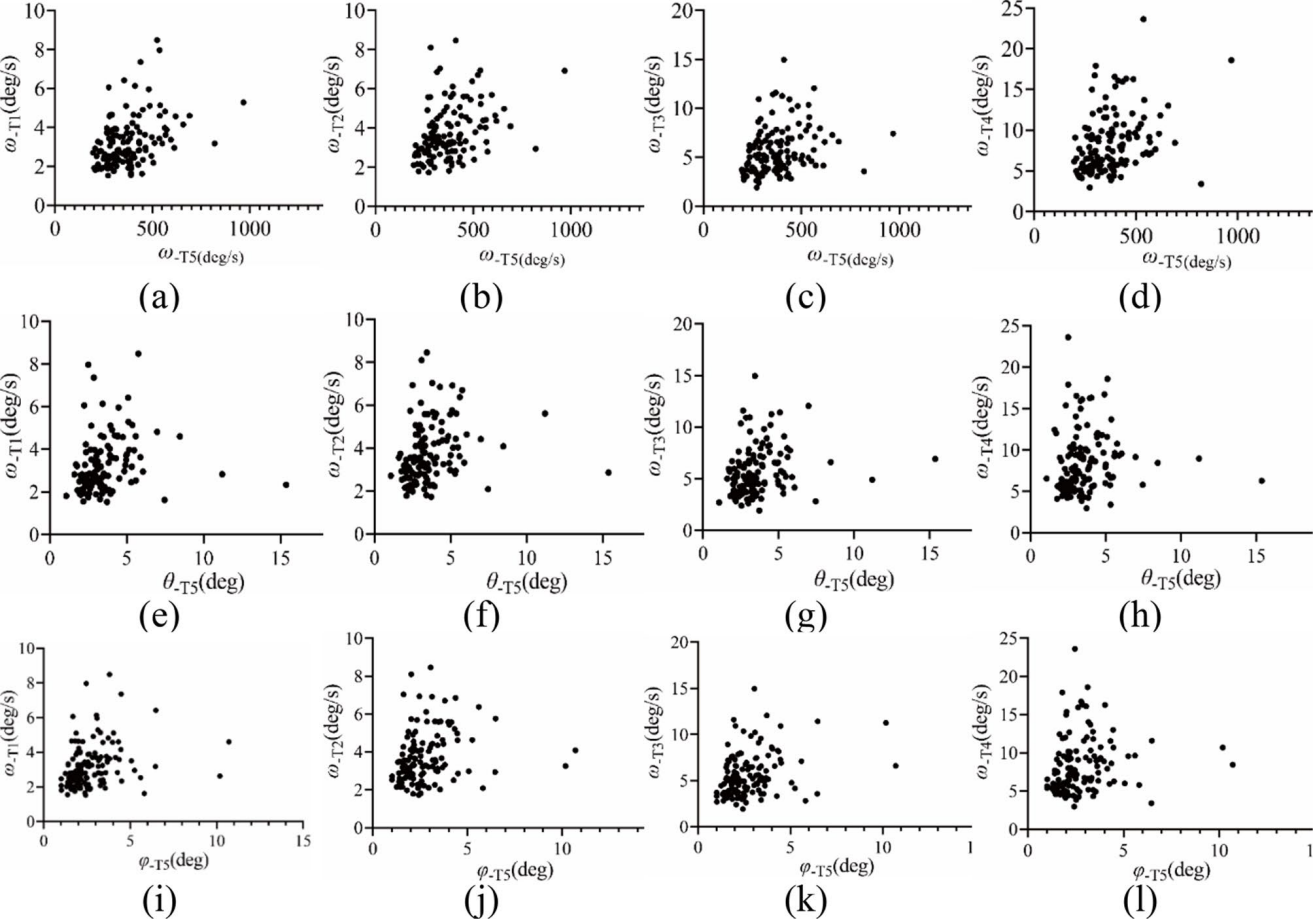
Scatter diagram of the correlation between dynamic and static balance in preschool children aged 4–5. Note: *ω*_−T5_ is the angular velocity modulus when walking on the balance beam, in deg/s; *θ*_−T5_ and *φ*_−T5_ are the pitch and roll angles when walking on the balance beam, in deg. *ω*_−T1_–*ω*_−T4_ represents the angular velocity modulus values of T1, standing with eyes open; T2, standing with eyes closed; T3, standing with eyes open on a foam pad; and T4, standing with eyes closed on a foam pad, in deg/s

A scatter diagram of the correlation between static and dynamic balance is shown in Fig. 2, and the test results are presented in Table 2. The results show that there is no correlation between the *t* and static balance, *ω*_−T1_–*ω*_−T4_ (*r*=-0.02–0.05, *P* = 0.566 − 0.999), and there is also no correlation between *t* and attitude angle (*r*=-0.08–0.10, *P* = 0.270–0.882). The correlation between *ω*_−T5_ and static balance *ω*_−T1_–*ω*_−T4_ (*r* = 0.19–0.27, *P* = 0.004–0.030) and that between *ω*_−T5_ and attitude angle (*r* = 0.02–0.23, *P* = 0.011–0.793) were both weak. When walking on the balance beam, the change in the body posture angle was weakly correlated with both the angular velocity modulus of the static balance (*r* = 0.18–0.33, *P* = 0.001–0.041) and the change in posture angle in static balance (*r*=-0.05–0.28, *P* = 0.001–0.886).

The results show that the relationship between static and dynamic balance is weak when body shaking speed and amplitude are used to reflect static and dynamic balance. When dynamic balance is reflected by walking on a balance beam, static balance is not related to dynamic balance. In general, the correlation between static and dynamic balance in children of this age group was weak.

**Table 2:** Correlation analysis of static and dynamic balance in preschool children aged 4–5

Static balance		Dynamic balance							
		*t*		*ω*_−T5_		*θ*_−T5_		*φ*_−T5_	
		*r*	*P*	*r*	*P*	*r*	*P*	*r*	*P*
	*ω*_−T1_	0.05	0.566	0.27	0.002	0.31	< 0.001	0.32	< 0.001
	*ω*_−T2_	0.01	0.999	0.25	0.004	0.33	< 0.001	0.29	0.001
	*ω*_−T3_	-0.02	0.819	0.19	0.030	0.26	0.003	0.18	0.041
	*ω*_−T4_	0.02	0.817	0.20	0.025	0.22	0.015	0.19	0.036
	*θ*_−T1_	0.10	0.270	0.20	0.021	0.19	0.037	0.12	0.174
	*θ*_−T2_	0.08	0.391	0.19	0.028	0.28	0.001	0.09	0.340
	*θ*_−T3_	0.03	0.779	0.08	0.379	0.13	0.138	0.05	0.578
	*θ*_−T4_	0.03	0.748	0.23	0.011	0.26	0.004	0.19	0.032
	*φ*_−T1_	0.03	0.778	0.18	0.046	0.24	0.007	0.24	0.006
	*φ*_−T2_	-0.08	0.373	0.07	0.452	0.15	0.086	0.14	0.113
	*φ*_−T3_	-0.01	0.882	0.02	0.793	0.11	0.219	0.01	0.886
	*φ*_−T4_	0.05	0.585	0.16	0.068	0.16	0.065	0.19	0.035

*ω*_−T1_ – *ω*_−T4_ represents the angular velocity modulus values of T1, standing with eyes open; T2, standing with eyes closed; T3, standing with eyes open on a foam pad; and T4, standing with eyes closed on a foam pad, in deg/s. *θ*_−T1_ –*θ*_−T4_ and *φ*_−T1_ – *φ*_−T4_ represents the pitch and roll angles of T1 to T4, in deg. *t* is the time taken to walk on the balance beam, in seconds. *ω*_−T5_ is the angular velocity modulus when walking on the balance beam, in deg/s; *θ*_−T5_ and *φ*_−T5_ are the pitch and roll angles when walking on the balance beam, in deg

## Discussion

By measuring the speed and amplitude of the COM while standing upright and walking on the balance beam, this study found that there was little relationship between static and dynamic balance in preschool children aged 4–5 years.

The weak relationship between the static and dynamic balance in children aged 4–5 years is consistent with the results of studies on people aged 6 years and over [5, 22, 23]. The reasons for the relatively small relationship between the two may be as follows. First, the neurophysiological structures involved in the superior central system are different. The EEG activity of the human body, when the participant is standing, is different from when the participant is walking. The cerebral cortex is more active while standing and the connection of the sensory-motor cortex is significantly weakened while walking, with the spinal cord neural network playing an important role [24]. Second, the neuromuscular mechanisms underlying task execution differs between the two situations. While standing, the human body relies more on reflex activity, requiring only a small amount of neuromuscular activity, whereas postural control during movement requires greater neuromuscular activity [25]. Third, the difficulty level of the two tasks also differs between the two situations. While standing statically, the body support points were stable, and only the COM moved within a small range. When walking on the balance beam, both the COM and support point move, and the COM remains stable when the support point moves. The tasks of preschool children aged 4–5 is complex and challenging [5]. Fourth, attention allocation differed while completing the two tasks. As compared to standing, walking requires higher attention [26], which may be more obvious when preschool children aged 4–5 complete the relatively difficult task of walking on a balance beam. The relationship between static and dynamic balance is more obvious than that in fiction; it reflects two specific tasks rather than a general ability [8]. This finding indicates that in the initial stage of balanced development, the two cannot replace and predict each other, suggesting that they need to be treated separately while recognizing, evaluating, and developing the balance of preschool children.

An additional finding of this study is that the relationship between the time taken to walk on the balance beam and the shaking amplitude of the COM when walking on the balance beam is small (the time indicator weakly correlates with the amplitude in the longitudinal direction, but does not correlate with the amplitude of the transverse direction shaking). That is, the correlation coefficient of the two indicators reflecting the same variable is small, which may be because the time indicator and the body shaking indicator show different abilities. This finding suggests that, under the condition of imperfect dynamic balance test indicators, it may be necessary to show the development level through multiple indicators. Nevertheless, there were some limitations in the present study. We recognize that due to the young age of the sample, participant attention may affect the performance of children. In addition, we did not conduct tests by randomly selecting items, but in the order of T1 to T5, which may impact the accuracy of the test results; however, we did not observe the influence of heteroscedasticity on the results. Moreover, the balance types were not sufficiently comprehensive. In addition to static and dynamic balance, balance can also be classified into three or four types. Given the particularity of preschool children and the fact that the test methods for other types of balance are not perfect, this study only discussed the relationship between static and dynamic balance and can continue to explore the correlation between the two, as well as active and reaction balance, in the future.

## Conclusions

The study results showed that the relationship between static and dynamic balance is relatively weak. Theoretically, it is helpful to further understand the relationship between the physical development, movement development, balance, and other abilities of preschool children, and aims to prevent children from falling and promoting rehabilitation. The use of different intervention contents and exercise methods to develop static and dynamic balance, respectively, are suggested. The use of only one test method when evaluating balance in children should be avoided and should at least include static and dynamic balance. Comprehensive calculations of static and dynamic balance were avoided when obtaining the total balance score.

## Data Availability

The database used to carry out this work is in the possession of the authors and will be provided to whoever requests it. Please contact Ruqiang Liu to obtain the data.

## Abbreviations

COM: centers of mass
mCTSIB: modified clinical test of sensor interaction and balance
ω: angular velocity modulus
θ: pitch
φ: roll
ψ: yaw
t: time of walking on the balance beam
